# Ground Glass Opacities Observed in a 26-Year-Old Coronavirus Disease 2019 (COVID-19) Rule-Out Patient With a History of Vape Use

**DOI:** 10.7759/cureus.10302

**Published:** 2020-09-07

**Authors:** Vivek N Patel, Michael Rouse, Christopher Brown, Sahil Pandya

**Affiliations:** 1 Internal Medicine, The University of Kansas Medical Center, Kansas City, USA; 2 Pulmonary and Critical Care Medicine, The University of Kansas Medical Center, Kansas City, USA

**Keywords:** e-cigarette and vaping product use associated lung injury (evali), covid, ground-glass opacity, ground glass opacity

## Abstract

Pulmonary imaging findings in e-cigarette and vaping use associated lung injury (EVALI) and coronavirus disease 2019 (COVID-19) may be similar. One such pulmonary radiographic finding is ground glass opacities (GGOs). These GGOs present a wide differential that is narrowed down through diagnostic testing, deliberation of past medical history as well as medication use, and social history. This case presents GGOs observed in a COVID rule-out admission clinically correlated with EVALI.

## Introduction

The objective of this case report is to describe a case of suspected e-cigarette and vaping use associated lung injury (EVALI) in the setting of the coronavirus disease 2019 (COVID-19) pandemic. Testing for COVID-19 may be performed on the basis of clinical suspicion if not universally performed in the setting of a hospital admission. GGO’s (ground glass opacities) are nonspecific radiologic findings associated with both EVALI and COVID-19 [1,2]. This case highlights the need to pursue diagnostic testing for ruling out COVID-19 when presented with a patient having correlative symptoms and the importance of detailed history taking when clinically correlating current vape use to EVALI.

## Case presentation

**Figure 1:**
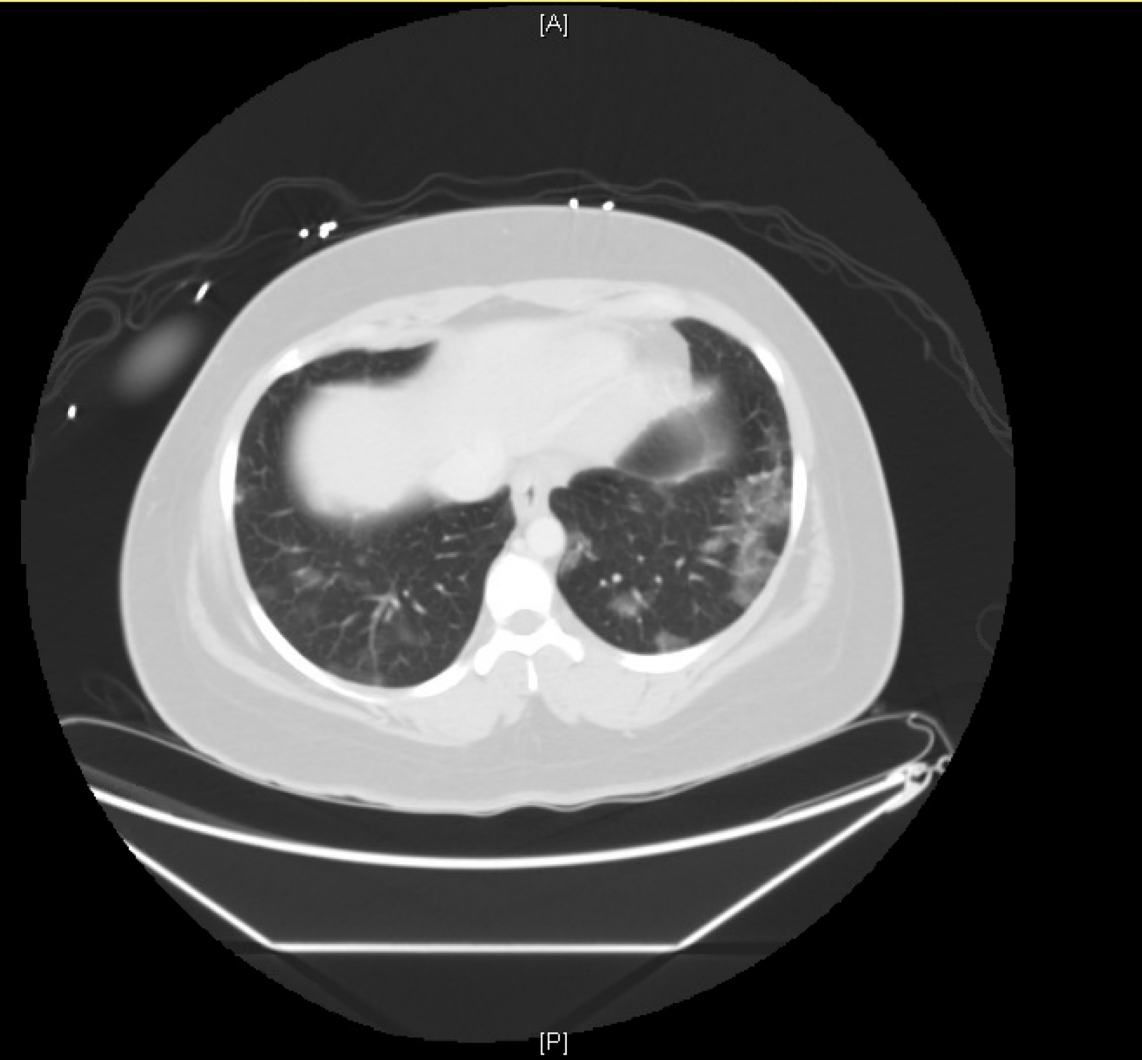
CT Abdomen/Pelvis basilar lungs demonstrating peripheral ground glass opacities (GGOs).

A 26-year-old female with a history significant for idiopathic chronic abdominal pain, diarrhea, cough, and daily marijuana vaping use presented to the emergency department with acute onset nausea, vomiting, and abdominal pain not controlled with her home medications of Prilosec and Compazine. She denied fever, chills, myalgias, or sore throat. She denied sick contacts, however, she did not follow the state-mandated stay at home order, social distancing recommendations, and frequently visited her close friends. Review of systems was pertinent for chronic non-productive cough unchanged with acute illness, abdominal pain, chronic unchanged diarrhea, nausea, vomiting, dizziness, and headache. She was afebrile with temperature of 36.7° Celsius, oxygen saturation of 99% on room air, blood pressure 145/95, heart rate at 105. Abdominal exam was remarkable for tenderness to palpation with voluntary guarding and the remainder of the physical exam, including pulmonary exam, was unremarkable. Associated lab findings included anion gap metabolic acidosis (19), elevated lactate (3.5), mild hyponatremia (133), leukocytosis (23.8), and lymphopenia (6). Dedicated chest imaging was not performed, but CT of the abdomen and pelvis was obtained. This imaging was negative for an acute inflammatory or obstructive process, but did show GGOs in bilateral lung bases (Figure 1). The patient was admitted for COVID-19 rule out and managed with fluid rehydration and symptom relief. COVID-19 polymerase chain reaction (PCR) nasopharyngeal swab testing was negative. Careful delineation of acute and chronic respiratory symptoms along with a negative COVID-19 test was key in arriving at the appropriate diagnosis. Overall impression of signs and symptoms were consistent with cannabinoid hyperemesis syndrome. Pulmonary findings of basilar GGOs were clinically correlated to EVALI attributed to ongoing vaping. Her symptoms improved and lab abnormalities were corrected with conservation management, and she was discharged the following day after vaping cessation education.

## Discussion

When approaching a patient with suspected COVID-19, characterizing symptoms is important. The most common presenting symptoms of COVID-19 in analysis of the Wuhan outbreak population were fever (43.8% on admission) and cough (67.8%). Diarrhea was noted rarely (3%). Most common lab findings include lymphocytopenia (83.2%), thrombocytopenia (36.2%), and leukopenia (33.7%) [1].

The most common radiologic finding on CT is GGOs present in 56.4% of patients with COVID-19 [1]. COVID-19 ranges from mild illness to sepsis with several levels of severity in between. Mild illness may have symptoms of sore throat, dry cough, fever, nasal congestion, malaise, myalgias, and headaches [3]. The finding of GGOs presents a broad differential, however the patient in this case did not have a history of opportunistic infections, connective tissue disease, or medications that are associated with pulmonary toxicity. 

Since COVID-19 pathology is variable and this patient had non-specific GGOs, precautions as COVID-19 under investigation were needed. The differential for ground glass opacities in COVID-19 settings must include EVALI should the patient give a correlating history. Most EVALI cases show basilar-predominant consolidation and GGOs [2]. There are no specific diagnostic criteria for EVALI, and other testable diagnoses must be ruled out before making the diagnosis in the clinical scenario of recent vaping or e-cigarette use [2]. 

## Conclusions

Amidst the cost of COVID-19 inpatient care and the number of rising EVALI cases since fall 2019, management attempts to promptly identify the disease process. Identification affects inpatient management, and treatment of these two disease processes may vary, especially with the accumulation of data derived from more cases and trials of pharmacotherapy. EVALI and COVID-19 are new disease processes that appear similarly and may each rapidly progress to acute hypoxic respiratory failure. Diagnostic uncertainty predicates detailed history taking for use of e-cigarettes or vaping instruments. Testing for COVID-19 is necessary to evaluate GGOs in the correct clinical setting, as implications for personal protective equipment (PPE) use and tailored discharge planning are paramount during a pandemic. This case highlights the dilemma created when alternate disease processes manifest in a similar clinical fashion to an ongoing pandemic. Successful management hinges on detailed history taking and systematic weighing of pre-test and post-test probability when approaching diagnostics.
